# Intracranial Hypotension After Surgery for Idiopathic Scoliosis Managed Conservatively: A Case Report and Literature Review

**DOI:** 10.7759/cureus.110921

**Published:** 2026-06-15

**Authors:** Masahiro Fukuda, Satoshi Suzuki, Kazuki Takeda, Kota Watanabe, Masaya Nakamura

**Affiliations:** 1 Department of Orthopaedic Surgery, Keio University School of Medicine, Tokyo, JPN

**Keywords:** conservative management, idiopathic scoliosis, late presentation of dural tear, postoperative cerebrospinal fluid leak, secondary intracranial hypotension

## Abstract

Intracranial hypotension (IH) following scoliosis surgery is a rare complication, most commonly associated with dural injury or pedicle screw malposition, often requiring revision surgery. We present a case of postoperative IH after surgery for scoliosis that was successfully managed with conservative treatment alone.

A 19-year-old female with idiopathic scoliosis underwent posterior spinal fusion from T2 to L1. The surgery was completed without intraoperative complications, including motor-evoked potential deterioration or dural injury. On postoperative day 3, she developed severe orthostatic headache accompanied by nausea and tinnitus. Cranial and spinal magnetic resonance imaging revealed diffuse dural thickening, pituitary enlargement, and epidural fluid collection consistent with IH. Computed tomography showed no definite malposition of pedicle screws. The patient was successfully treated with bed rest and hydration for one week without recurrence. This case highlights that revision surgery is not always necessary for IH following scoliosis surgery, especially in cases of minor medial pedicle screw breach or unrecognized dural tear.

## Introduction

Adolescent idiopathic scoliosis (AIS) is a three-dimensional spinal deformity affecting approximately 1% to 3% of adolescents [1]. Posterior spinal fusion using pedicle screw is a widely accepted standard treatment for progressive curves; however, pedicle screw misplacement and medial breach may still occur, sometimes resulting in neurological deficits or cerebrospinal fluid (CSF) leakage [2]. Even with the use of advanced technologies such as navigation and robotic assistance, these complications cannot be completely prevented [3].

Furthermore, most previously reported cases of intracranial hypotension (IH) following scoliosis surgery have been attributed to dural violation caused by misplaced pedicle screws and typically require reoperation for screw removal or dural repair [4,5]. IH occurring after AIS surgery without identifiable screw malposition or intraoperative dural tears is rare. Although conservative management with hydration is widely recognized as the standard treatment for spontaneous intracranial hypotension [6], there have been no previous reports of IH developing after AIS surgery and improving solely with conservative therapy.

Here, we report a case of intracranial hypotension in the absence of any identifiable screw malposition or dural tear and discuss the potential underlying mechanisms and management considerations.

## Case presentation

A 19-year-old female patient presented with a progressive spinal deformity. She was diagnosed with AIS at 12 years of age and initially managed with brace treatment. However, due to poor compliance, her curve gradually progressed, eventually reaching the surgical threshold by the age of 19 years. She had no significant past medical or family history relevant to spinal deformity progression.

Physical examination demonstrated a right thoracic hump without neurological abnormalities. Standing radiographs demonstrated a proximal thoracic Cobb angle of 41° from T1 to T6 and a main thoracic Cobb angle of 50° from T6 to T12. Bending radiographs showed that the proximal thoracic curve changed from 41° to 39°, and the main thoracic curve improved from 50° to 35°, leading to the diagnosis of a structural double thoracic curve (Fig. 1). Preoperative magnetic resonance imaging (MRI) revealed no pathological abnormalities, including Chiari malformation.

**Figure 1:**
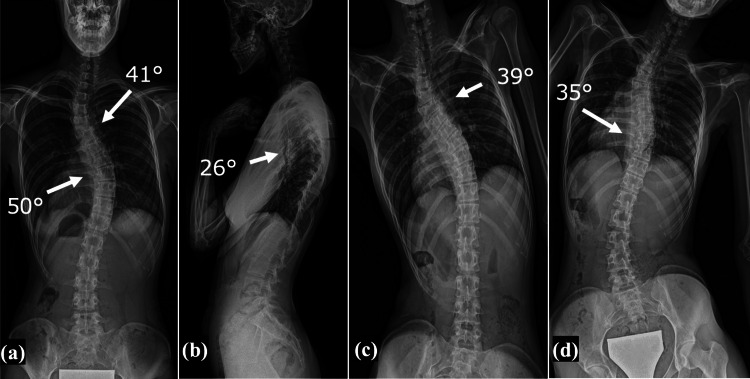
Preoperative Radiograph Preoperative standing posteroanterior radiographs (a, b) showing the Cobb angles of the proximal thoracic and main thoracic curves (41° and 50° (arrows))  with normal thoracic kyphosis (26° (arrow)). Side-bending radiographs (c, d) demonstrating rigid scoliosis, consistent with a structural double thoracic curve (arrows).

Posterior spinal fusion from T2 to L1 was performed. After soft tissue and muscular exposure, pedicle screws were inserted at all planned levels, except for the right T4 and T5 pedicles, where screw insertion was avoided because of the narrow pedicle diameter. Following Ponte osteotomies from T2 to T12, Nesplon® tape Nesplon tape (ultra-high-molecular-weight polyethylene tape; Alfresa Pharma Corporation, Osaka, Japan)** **was applied around the laminae of right T4 and T5. Deformity correction was achieved using rod rotation and translation techniques. The operative time was 3 hours and 50 minutes, and the estimated blood loss was 170 mL. No intraoperative complications, including motor-evoked potential (MEP) deterioration or dural injury, were observed.

She began ambulation on postoperative day 2. On postoperative day 3, she developed a severe orthostatic headache that worsened in the upright position and improved when lying supine. The headache was accompanied by nausea and tinnitus and was unresponsive to the analgesics. Brain MRI demonstrated diffuse dural thickening and pituitary enlargement. These findings were consistent with intracranial hypotension (IH) (Fig. 2a, 2b).

**Figure 2:**
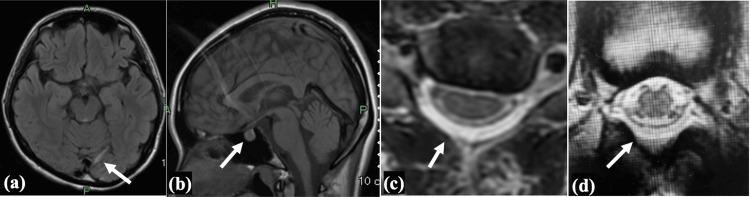
Postoperative Magnetic Resonance Imaging Brain magnetic resonance imaging (MRI) demonstrating diffuse dural thickening (arrow) and pituitary enlargement (arrow), consistent with intracranial hypotension (a, b). Spinal MRI showing extensive dorsal epidural cerebrospinal fluid accumulation (arrow) extending from the cervical to the thoracolumbar spine (c, d).

Spinal MRI revealed extensive cerebrospinal fluid (CSF) accumulation in the dorsal epidural space, extending from the cervical to the thoracolumbar spine (Fig. 2c, 2d). Although axial computed tomography raised suspicion of a possible medial breach of the left pedicle screws at T6-T9 (Fig. 3a), coronal images demonstrated no definite screw malposition (Fig. 3b).

**Figure 3:**
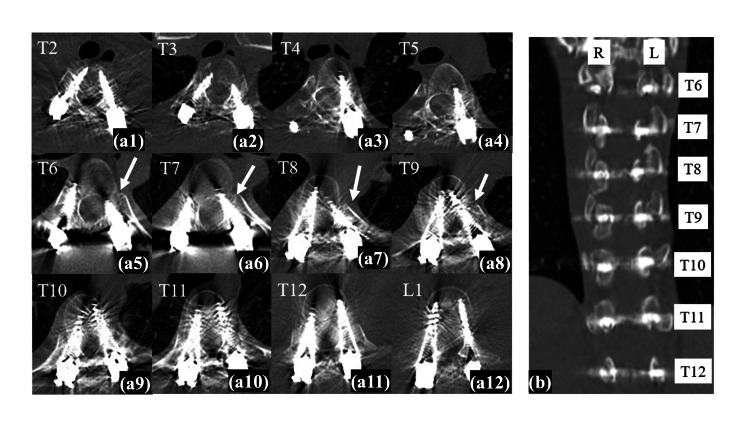
Postoperative CT CT images a1-12 show postoperative axial CT images from T2 to L1. Postoperative axial CT showed that the left pedicle screws (arrows) at T6–T9 appeared to be slightly directed medially (a5-8), whereas coronal images showed no definite screw malposition (b).

Based on the clinical presentation and imaging findings, postoperative IH was diagnosed. Because of the absence of a clear CSF localized leak point and definite screw malposition, the initial management consisted of one week of conservative treatment, including bed rest and an intravenous fluid intake of 1.5 litres per day. If no clinical improvement was observed, the next plan was to proceed to radionuclide cisternography or myelography; however, she began ambulation on postoperative day 10 without recurrence of headache and was discharged on postoperative day 20. Follow-up MRI at one year demonstrated complete resolution of the epidural CSF collection (Fig. 4a, 4b). At the final follow-up three years postoperatively, she maintained favorable spinal alignment (Fig. 4c).

**Figure 4:**
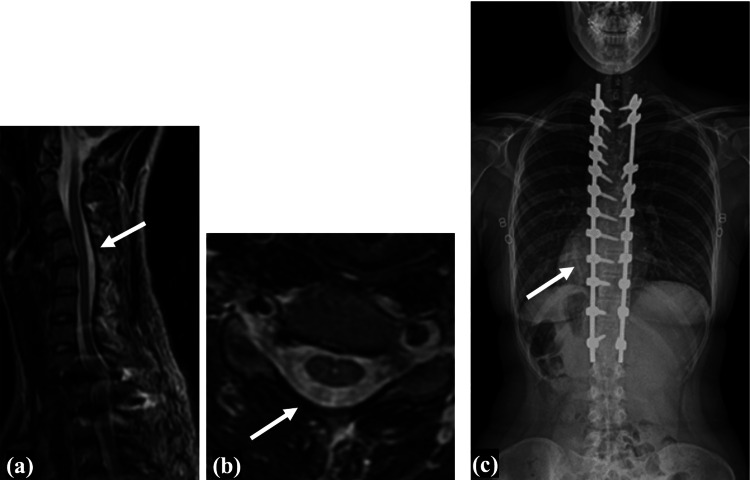
One-year postoperative MRI One-year postoperative MRI showing complete resolution of the epidural cerebrospinal fluid (CSF) collection (arrows) with no recurrence of CSF leak (a, b). At the three-year follow-up, the patient demonstrated maintained spinal balance (c (arrow))

## Discussion

Intracranial hypotension (IH) results from cerebrospinal fluid (CSF) leakage and typically presents with severe orthostatic headaches, nausea, and auditory symptoms. As for the mechanism of the symptoms, CSF leakage causes downward displacement of the brain and traction on pain-sensitive intracranial structures such as cerebral vessels, cranial nerves, and the skull base [7]. IH can be classified as primary, referred to as spontaneous intracranial hypotension, which is most often caused by spontaneous spinal CSF leaks or secondary to spinal puncture, shunt over-drainage, or spinal trauma [7,8]. Although several spinal pathologies have been reported to cause IH, including degenerative conditions with osteophytes, herniated discs, and cystic spinal lesions [9,10], IH related to spine surgery has been commonly associated with intraoperative dural tears or pedicle screw misplacement [4-5,11-12]. 

Posterior spinal fusion using pedicle screws is the gold standard for scoliosis surgery; therefore, most cases of intracranial hypotension after scoliosis surgery have been attributed to screw malposition. To the best of our knowledge, 11 cases of intracranial hypotension caused by pedicle screw malposition have been reported to date (Table 1).

**Table 1:** Summary of Reported Cases of Intracranial Hypotension Following Scoliosis Surgery

Author	Year	Cases	Cause	Treatment	Reference
Gilmour GS	2019	1	Intraoperative dural injury	Reoperation (dural repair)	[13]
Albayram S	2008	1	Misplaced pedicle screw	Reoperation (screw removal and dural repair)	[4]
Nowak R	2011	1	Misplaced pedicle screw	Reoperation (screw removal and dural repair)	[14]
Heiler U	2022	1	Misplaced pedicle screw	Reoperation (screw removal and dural repair)	[15]
Floccari L	2017	4	Misplaced pedicle screw	Reoperation (screw removal and dural repair)	[5]
Dede O	2014	1	Misplaced pedicle screw	Reoperation (screw removal and dural repair)	[16]
Mac-Thiong JM	2013	2	Misplaced pedicle screw	Reoperation (screw removal)	[12]

Albayram et al. reported a 14-year-old girl who developed severe orthostatic headaches three months after surgery for AIS, in whom a thoracic pedicle screw was found to penetrate and traverse the dura at the T3-T4 level, resulting in a CSF leak and intracranial hypotension [4]. Mac-Thiong et al. reported nine cases of pedicle screws placed entirely within the spinal canal after AIS surgery, two of which developed early postoperative orthostatic headaches attributable to CSF leakage, underscoring that medially misplaced screws can produce symptoms consistent with intracranial hypotension [12]. Floccari et al. identified four patients out of 322 AIS cases (0.9%) who presented with delayed orthostatic headaches secondary to CSF leakage. Persistent CSF extravasation caused by malpositioned pedicle screws was identified in all patients, with one demonstrating severe malposition and the remaining three showing ≤2 mm medial breach [5]. Cases of convulsive status epilepticus and massive acute intracranial subdural hematoma have been reported as secondary complications of intracranial hypotension resulting from screw-related dural violation [13,14]. Although the prognosis is favorable, all previously reported cases required reoperation, including screw removal and dural repair. To our knowledge, this is the first report of intracranial hypotension after scoliosis surgery that resolved with conservative treatment alone without revision surgery

One of the most plausible explanations for IH in this case is an undetectable minor pedicle screw breach, as a small medial violation may have occurred but remained undetected owing to the inherent limitations of CT imaging. Another possible mechanism is an unrecognized intraoperative dural tear, potentially caused by dural injury during passage of the Nesplon tape beneath the right T4 and T5 laminae or during the Ponte osteotomy. A further explanation is underlying connective tissue fragility, whereby subtle connective tissue weakness may have rendered the dura more susceptible to microinjury during deformity correction. Despite these possibilities, the patient achieved complete clinical and radiographic recovery with conservative therapy alone, without the need for an epidural blood patch or revision surgery. Although this is a single case report, this case highlights that revision surgery is not always necessary for IH following scoliosis surgery, especially in cases of minor medial pedicle screw breach or unrecognized dural tear.

## Conclusions

We report a case of IH following scoliosis surgery that was successfully managed with conservative treatment alone. Revision surgery is not always necessary for IH following scoliosis surgery, especially in cases of minor medial pedicle screw breach or unrecognized dural tear.
